# High Life Expectancy of Bacteria on Lichens

**DOI:** 10.1007/s00248-016-0818-5

**Published:** 2016-07-27

**Authors:** Tomislav Cernava, Gabriele Berg, Martin Grube

**Affiliations:** 1 Institute of Environmental Biotechnology, Graz University of Technology, Petersgasse 12, 8010 Graz, Austria; 2 Institute of Plant Sciences, University of Graz, Holteigasse 6, 8010 Graz, Austria

**Keywords:** Herbarium, Storage, Thallus, Survival, Lichen-associated bacteria, *Lobaria pulmonaria*

## Abstract

Self-sustaining lichen symbioses potentially can become very old, sometimes even thousands of years in nature. In the joint structures, algal partners are sheltered between fungal structures that are externally colonized by bacterial communities. With this arrangement lichens survive long periods of drought, and lichen thalli can be revitalized even after decades of dry storage in a herbarium. To study the effects of long-term *ex situ* storage on viability of indigenous bacterial communities we comparatively studied herbarium-stored material of the lung lichen, *Lobaria pulmonaria*. We discovered that a significant fraction of the lichen-associated bacterial community survives herbarium storage of nearly 80 years, and living bacteria can still be found in even older material. As the bacteria reside in the upper surface layers of the lichen material, we argue that the extracellular polysaccharides of lichens contribute to superior life expectancy of bacteria. Deeper understanding of underlying mechanisms could provide novel possibilities for biotechnological applications.

## Introduction

Lichens are widely known as self-sustaining symbioses of fungi and algae (including cyanobacteria), which develop compound structures, the lichen thalli. In most lichens the photosynthetic partners are sheltered between peripheral fungal layers to optimize the algal function as providers of fixed carbohydrates to the symbiotic community [1]. Lichen thalli are environmentally robust and able to survive periodic desiccation, extremes of temperatures and even outer space conditions [2]. Research of the past ten years revealed the diversity of lichen-associated bacteria [3, 4], which appear as a third component in this symbiosis. Bacteria assemble in host-specific communities and cover lichen thalli in a biofilm-like manner [5]. Previous studies demonstrated that the composition of the bacterial communities is independent from seasonal variation [3]. Although only a minor fraction of the lichen-associated bacteria can be cultured, bacteria have even been isolated after storage and long-distance transport of the thalli [6]. Both of these latter findings suggest that bacterial communities are little altered by climatic conditions and have potentially high survival rates on the lichen thalli.

Lichen diversity is traditionally documented and archived with dried herbarium specimens, and these are also a mainstay of taxonomic research. Preliminary microscopic studies show that bacterial colonies are also present in herbarium material of lichens, and that the colonization patterns are not different from freshly collected samples. In this context, it is also known that isolated algal and fungal partners can be cultivated from herbarium material after decades of herbarium storage ([7], E. Stocker pers. comm.). We argue that lichen-associated bacteria have similarly high survival rates when the material is maintained in dried conditions but this hypothesis has not been studied. In this short communication we report the first quantitative assessment of bacterial survival on herbarium specimens of the lung lichen, *Lobaria pulmonaria* (L.) Hoffm. *L. pulmonaria* is a common bioindicator of forest continuity and present in habitats that are almost completely devoid of pollutants (in particular SO_2_). In these habitats, this species develops large and easily recognized thalli, which have regularly been collected and stored to document dynamics of air pollution, land use, or climate change over times.

## Material and Methods

### Herbarium Specimens

Small fragments of lichen thalli were removed under sterile conditions from herbarium specimens stored in the herbarium of the University of Graz (GZU). The specimens were collected in August 2012 (Austria, Styria, Johnsbachtal, Untere Koderalm, coll. M Grube), June 2004 (Austria Styria, Tamischbachgraben, coll. P. Bilovitz), and June 1937 (Austria, Styria, Johnsbachtal, coll. K Schittengruber).

### Differential Visualization of Bacteria on Lichen Surfaces

Fragmented *L. pulmonaria* samples from the herbarium of the University of Graz (GZU) were stained with the LIVE/DEAD® Baclight kit^TM^ (Molecular Probes). The imaging was performed with a confocal laser scanning microscope (Leica TCS SPE confocal microscope, Leica Microsystems). Excitation wavelengths of 488 and 532 nm were used for the SYTO® 9 and propidium iodide fluorescent dye respectively. The light emission was detected in the range of 496–560 nm for SYTO® 9 and 600–680 nm for propidium iodide. Settings for photomultiplier gain and offset were adjusted to obtain an optimal signal/noise ratio. The confocal stacks were merged to obtain a maximum projection of all channels.

### Differential Quantification of Bacteria in Herbarium Samples

A qPCR-based approach was utilized to determinate the gene copy number of the Unibac-II-515f/Unibac-II-927r fragment. Herbarium samples of *L. pulmonaria* were fragmented and washed in 20 ml PBS (7 g/l Na_2_HPO_4_, 3 g/l KH_2_PO_4_, 4 g/l NaCl; pH, 7–7.2) for 30 min at 300 rpm. The suspension was transferred into four 2 ml reaction tubes for each sample. Two reaction tubes for each sample were supplemented with propidium monoazide (PMA; GenIUL) to a final concentration of 20 μg/ml. All samples were incubated on ice in the dark while shaking at 100 rpm for 50 min. The tube lids were then opened after incubation and placed under a LED light source for activation of PMA with an emission maximum of 520 nm for 10 min. PMA forms covalent bonds with available DNA but cannot pass through undisrupted cell membranes [8]. This step excludes DNA from dead cells in further analyses. The samples were centrifuged at 13,500 rpm, 4 °C for 15 min and the supernatants were discarded. Thereafter, the pellets from the same samples and treatments were resuspended in 500 μl PCR-grade water. After a second centrifugation at 13,500 rpm, 4 °C for 15 min the supernatants were discarded and the pellets were further utilized for DNA extraction with the PowerSoil DNA extraction kit (MoBio Laboratories Inc.). Quantification of 16S rDNA fragments from the DNA extract and standards containing the Unibac-II fragments were prepared according to [9]. The sample obtained in 2012 was diluted 1:10 due to results from pre-analyses while the other two samples were not diluted. The KAPA SYBR FAST qPCR Kit (Kapa Biosystems) was used on the Rotor Gene 6000 (Corbett Research) to obtain gene copy numbers for the specific fragments in three replicates. The respective sample weights and dilution factors were used to calculate the gene copy numbers/g lichen dry weight.

## Results and Discussion

**Fig. 1:**
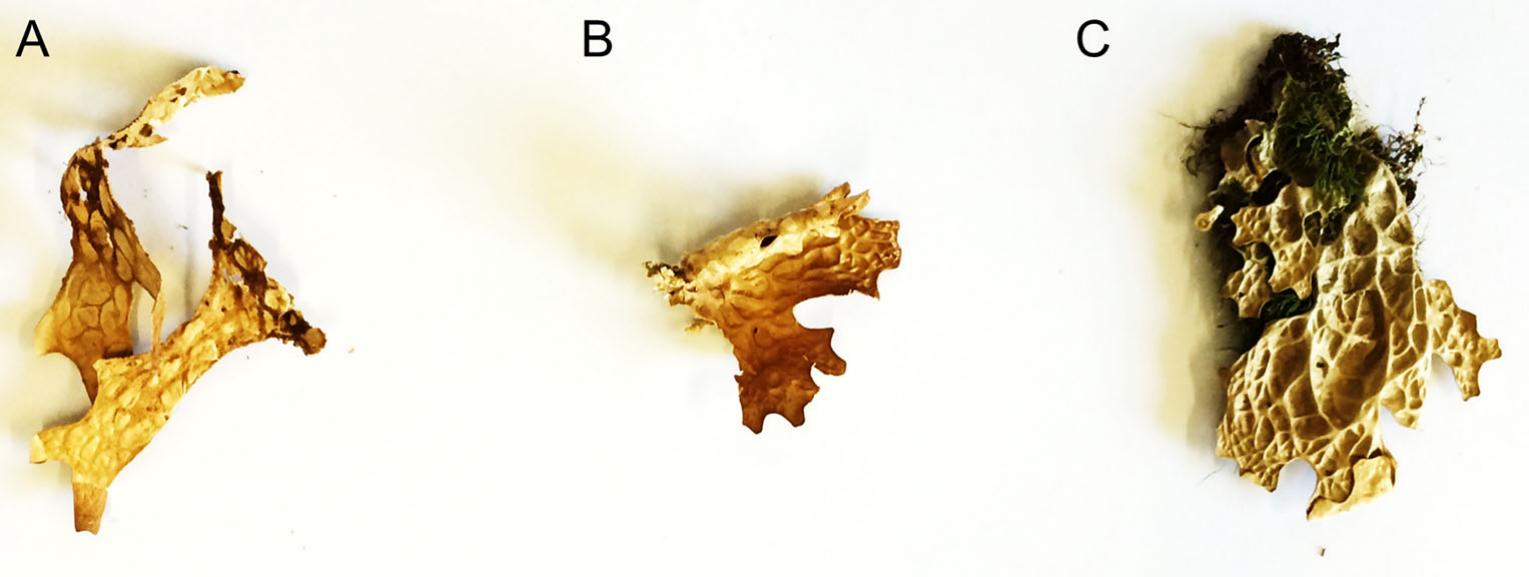
Herbarium specimens of *Lobaria pulmonaria*. **a** from 1937, **b** from 2004, **c** from 2012 (further information in the text)

**Fig. 2:**
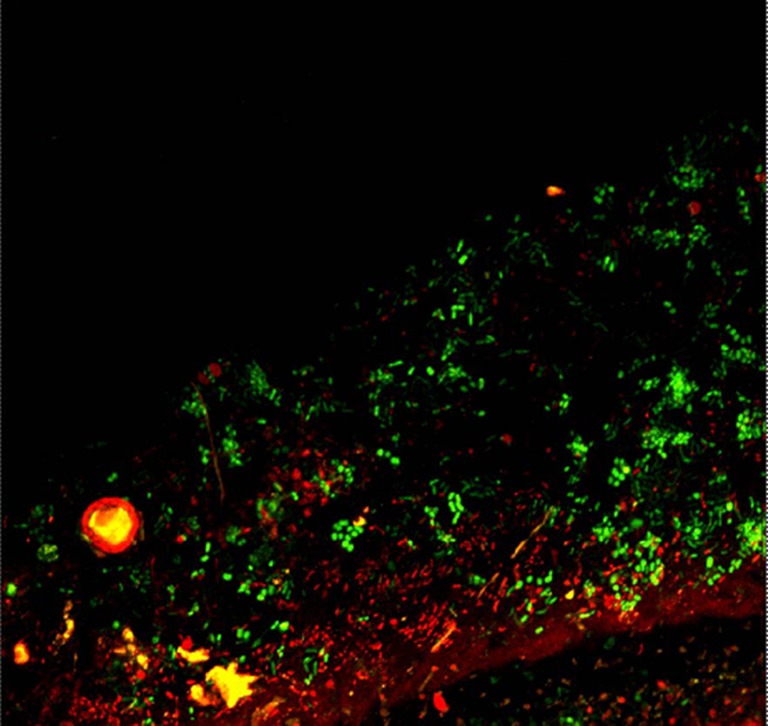
Confocal laser scanning image of LIVE/DEAD staining of the microbial community on the upper surface of *Lobaria pulmonaria* (sampled 2012; image obtained by M. Cardinale). Living bacteria with a functional cell membrane are stained in *green*, while dead bacteria with an impaired cell membrane are stained in *red*

**Fig. 3:**
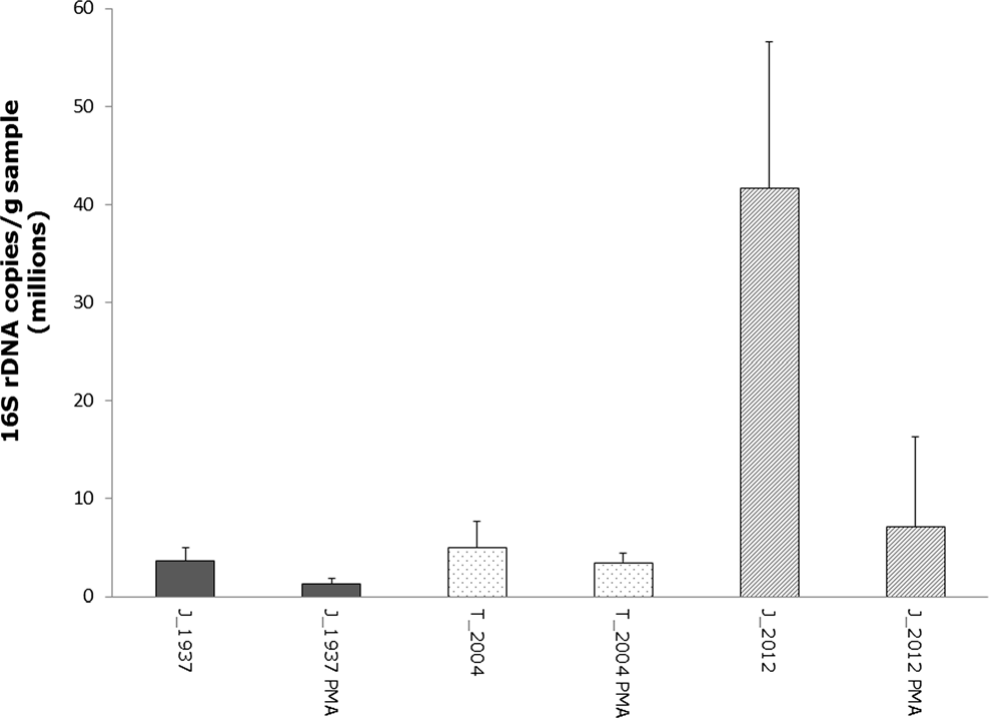
qPCR-based quantification of bacteria in herbarium samples. The bacterial fractions were washed from lichen surfaces and quantified with and without addition of PMA. The labels include the original sampling site (*J* Johnsbach, *T* Tamischbach) and the year of the sampling

The present data provide the first evidence that bacterial cells are able to survive long-term herbarium storage of lichens and provide a first insight into the survival rates of bacteria on lichen thalli. The specimens were kept dark and dry in paper envelopes and retained their original shape (Fig. 1). However, some bleaching of the specimens was observed. We observed that a fraction of the lichen-associated bacteria is dead already on thalli stored for 2 years, according to live/dead staining (Fig. 2), yet, qPCR results suggest that a considerable fraction of bacteria survive on thallus surfaces for at least 80 years (Fig. 3). The 16S rDNA gene copy number was quantified in a differential approach to access living bacteria. Overall a decrease of living bacteria on the thalli may be observed, even though one of the specimens from 2012 exhibited an unexpectedly high load of living bacteria (mean value of 7.1 × 10^6^ gene copies/g sample). Correspondingly, a correlation analysis did not indicate a linear or exponential trend in our data. The second highest number was observed for the sample collected in 2004 (mean value of 3.5 × 10^6^ gene copies/g sample), followed by the sample from 1937 (mean value of 1.3 × 10^6^ gene copies/g sample). The range for untreated samples was between 3.7 × 10^6^ and 4.2 × 10^7^ gene copies/g sample (mean values). In this context, it is noteworthy to mention that live/dead staining might be biased by specific properties of the utilized fluorophores as well specific biotic parameters, e.g. the presence of biofilms [10]. The present study combines two independent methods to access the viability of colonizing bacteria, but further exploration is still required. Further approaches include a spanning isolation of cultivable bacteria from herbarium material and also next-generation sequencing-based approaches.

The role of apparently dead bacteria on the lichen thalli needs further research and may add novel aspects to the discussion of bacterial functions in the lichen symbiosis. So far, functional assays with culture isolates, as well as metagenomics and metaproteomic analyses suggest a multitude of bacterial functions in lichens [3, 4, 11]. Usually, bacteria are evenly distributed on the thallus in a biofilm-like manner [5], with *Proteobacteria* as the most abundant group. A non-surviving fraction of the bacteria could represent an additional source of nutrients for the symbiotic partnership and at least a part of the dead bacteria might originate from an unspecific bacterial “rain” from the environment. However, this hypothesis still needs to be tested by isotope-labelling approaches.

The discovery of substantial survival rates of bacteria on dried material also opens exciting further options of research with lichens as bio-resources. The utility of plant herbaria for applied research such as drug discovery was already emphasized recently by a note in The Lancet [12]. Forays of lichen diversity have been conducted since more than 200 years, including remote and hostile areas, which are even today difficult to access without substantial financial and logistic effort. We therefore suggest that herbarium material of lichens could be an interesting bio-resource of culturable bacteria from habitats of extreme latitude or altitude (such as Antarctica, the Himalayas). These bacteria may possess useful biosynthetic pathways for novel secondary metabolites or otherwise biotechnological interesting traits. We also think that herbaria largely maintain the original microbiome composition of the fresh lichens. The maintenance is facilitated because lichens are usually collected in (a) relatively dry conditions and (b) dry out faster than plants, which need much more care during herbarization to avoid moulding and bacterial degradation. The quick drying of lichens is due to the lack of a cuticula, as typically found in plants (except in lower plants such as mosses). Lichens loose water rapidly over their entire surface. With too low content of water, life processes are largely stopped and the microbial communities remain largely unaffected. These differences from plants also allow for extended time course analyses of lichen microbiota over many years. While the evolutionary dynamics of individual pathogenic bacterial strains has been studied with dried plants [13], the present observations may open the possibility and unique chance to track the dynamics and evolution of entire microbial communities on the same long-living lichen structures or in the same habitat using dry-stored biological material. While anticancer properties of lichen polysaccharides are known [14], other technical uses are so far unexplored. We assume that the polysaccharide matrix of lichens, in which the majority of lichen-associated bacteria is embedded, may delay degradation of bacteria. With this natural analogue of bacterial microencapsulation, lichens could add another aspect as a biotechnologically interesting resource, i.e. for utilization as a xeroprotectant.
